# Comprehensive Analysis of Immune Infiltrates of Ferroptosis-Related Long Noncoding RNA and Prediction of Colon Cancer Patient Prognoses

**DOI:** 10.1155/2022/9480628

**Published:** 2022-02-27

**Authors:** Wenzheng Chen, Yafei Chen, Li Liu, Yukang Wu, Pengcheng Fu, Yi Cao, Jianbo Xiong, Yi Tu, Zhengrong Li, Yi Liu, Zhigang Jie

**Affiliations:** ^1^Department of Gastrointestinal Surgery, The First Affiliated Hospital of Nanchang University, China; ^2^Second Abdominal Surgery Department, Jiangxi Province Cancer Hospital, China; ^3^School of Medicine, Southern University of Science and Technology, China; ^4^Medical College of Nanchang University, China; ^5^Department of Pathology, The First Affiliated Hospital of Nanchang University, China

## Abstract

Ferroptosis is a newly defined mode of programmed oxidative cell death. Knowledge of ferroptosis-related long noncoding (lnc) RNA in the tumor immune microenvironment of colon cancer is lacking. We systematically analyzed the correlations between ferroptosis-related lncRNAs and the tumor microenvironment, immune cell infiltration, and patient prognosis for 379 colon cancer samples in the Cancer Genome Atlas (TCGA). Using consensus clustering, we divided the 379 colon cancer patients into two subgroups (clusters 1 and 2) based on the differentially expressed ferroptosis-related lncRNAs. Cluster 1 was preferentially associated with longer overall survival, upregulated immune checkpoint inhibitor expressions, higher immunoscores, higher stromal scores, higher estimated scores, and distinct immune cell infiltration. Cancer- and metabolism-related pathways were enriched by gene set enrichment analyses. We constructed a prognostic signature of 15 ferroptosis-related lncRNAs (ZEB1-AS1, LINC01011, AC005261.3, LINC01063, LINC02381, ELFN1-AS1, AC009283.1, LINC02361, AC105219.1, AC002310.1, AL590483.1, MIR4435-2HG, NKILA, AC021054.1, and AL450326.1) and divided the patients into the high- and low-risk-score groups. The signature was validated using TCGA training and testing cohorts. The risk signature was an independent prognostic factor for predicting survival and excellently predicted the prognoses of patients with colon cancer. Moreover, the risk signature was related to immune characteristics. Chemosensitivity analyses showed that low-risk-score patients were more sensitive to sorafenib. In summary, our work revealed the important role of ferroptosis-related lncRNAs in the tumor microenvironment and immune cell infiltration and may help determine personalized prognoses and treatment for patients with colon cancer.

## 1. Introduction

As one of the most common malignant tumors worldwide, colon cancer exhibits high morbidity and mortality, and the age of onset is becoming increasingly younger in China [1]. According to Cancer Statistics, approximately 148,000 new cases of colon cancer were diagnosed in the United States in 2020, leading to more than 53,200 deaths [2]. Moreover, people are being diagnosed with colon cancer at increasingly younger ages, indicating that the incidence in younger people is gradually trending upward. To date, the precise underlying mechanisms of colon cancer and the initiation of its progression remain unclear. Ferroptosis is a newly defined mode of programmed oxidative cell death, which is distinct from other cell deaths such as apoptosis, necrosis, and autophagy. Ferroptosis is iron-dependent and is triggered by lipid peroxidation and lethal reactive oxygen species (ROS) [3]. Many studies have shown the roles of ferroptosis in gastrointestinal cancer progression, invasion, migration, and death. For example, exosomal miR-522 secreted by cancer-associated fibroblasts targets ALOX15 and blocks lipid-ROS accumulation to inhibit ferroptosis in gastric cancer cells [4]. The polyunsaturated fatty acid (PUFA) biosynthesis pathway was shown to play an essential role in ferroptosis and determine ferroptosis sensitivity in gastric cancer [5]. Betulaceae extract induced HO-1 expression and resulted in ferroptosis-associated cell death in human colon cancer cells [6]. Cytoglobin, a regulator of lipid ROS, promoted sensitivity to ferroptosis by regulating the p53-YAP1 axis in colon cancer cells [7].

Increasing evidence suggests that ferroptosis-related long noncoding (lnc) RNAs play important roles in tumorigenesis, progression, and metastasis via multiple mechanisms. The lncRNAs GABPB1-AS1 and GABPB1 can regulate erastin-induced ferroptosis in hepatocellular carcinoma cells [8]. The lncRNA LINC00336 inhibits ferroptosis in lung cancer by functioning as a competing endogenous RNA [9]. MT1DP can induce ferroptosis by regulating the miR-365a-3p/NRF2 axis in non-small cell lung cancer cells [10].

However, whether ferroptosis-related lncRNAs are correlated with the prognosis of patients with colon cancer remains unknown. Hence, we constructed and validated a ferroptosis-related lncRNA prognostic signature, and explored the potential mechanism in colon cancer. The tumor microenvironment, immune cell infiltration, immune check inhibitors, functional enrichment, and chemosensitivity were also analyzed. Investigating the effects of ferroptosis-related lncRNAs on tumor immune infiltration will help decode how the tumor microenvironment is modulated and help better predict the prognosis and treatment outcomes of patients with colon cancer.

## 2. Materials and Methods

### 2.1. Dataset Collection

Raw counts of RNA-seq transcriptome data and the corresponding clinical data for 39 normal colon tissues and 379 colon cancer tissues were extracted from TCGA database. Data for 259 ferroptosis-related genes were downloaded from FerrDb (http://www.zhounan.org/ferrdb) [11] (Table S1). Pearson's correlation was used to assess the relationship of the ferroptosis-related lncRNAs and colon cancer genes. A Pearson correlation coefficient > 0.3 and *p* < 0.01 were considered statistically significant. Prognostic ferroptosis-related lncRNAs were screened via the univariate Cox regression analyses.

### 2.2. Identification of Subgroups and Evaluation of Immune Infiltration

According to the expressions of the included ferroptosis-related lncRNAs, all colon cancer patients were divided into subgroups via the “ConsensusClusterPlus” package [12]. Survival analysis and gene set enrichment analyses (GSEA) between two subgroups were also performed. ESTIMATE [13] algorithms were used to evaluate the immune, stromal, and estimated scores. Infiltration data for 22 immune cells were downloaded from the TIMER [14] and CIBERSORT [15] databases. The relationships between the expression levels of genes related to immune check inhibitors and their subgroups, including ferroptosis-related lncRNAs, were also studied.

### 2.3. Construction and Validation of the Ferroptosis-Related lncRNA Prognostic Signature

The prognostic signature of 15 ferroptosis-related lncRNAs was constructed using least absolute shrinkage and selection operator (LASSO) regression analysis. The formula for the prognostic signature was

(1) $$\begin{array}{c} \text{risk score}=\sum \left(\text{Exp}\left[\text{lncRNA}\right]\times \text{coef}\left[\text{lncRNA}\right]\right), \end{array}$$

where Exp(lncRNA) is the corresponding expression of the included lncRNA and coef (lncRNA) represents the regression coefficient. The patients were randomly divided into the training or testing cohort. According to the above formula, the risk score of each patient was separately calculated for the training and testing cohorts. The patients were further classified into low-risk or high-risk groups according to the median risk score.

Survival analysis and univariate and multivariate Cox regression analyses were conducted to verify the independent prognostic value of the ferroptosis-related lncRNA prognostic signature. Nomograms that included age, sex, stage, Tumor, Node, Metastasis (TNM) classification, and risk score were used to calculate the total score and predict the 1-, 3-, and 5-year survival probabilities. The 1-, 3-, and 5-year dependent receiver operating characteristic (ROC) curves were used to assess the nomogram performance.

### 2.4. Application of the Signature in Clinical Treatment

To evaluate the use of the signature in clinically treating colon cancer, we calculated the half inhibitory concentrations (IC50s) of commonly administered chemotherapeutic and target therapeutic drugs, including cisplatin, paclitaxel, sorafenib, and sunitinib, with the pRRophetic package [16]. The difference in the IC50s between the high- and low-risk groups was compared via the Wilcoxon signed-rank test, and the results are shown as box plots.

### 2.5. Statistical Analysis

All statistical analyses were performed in R, version 4.0.4. Differentially expressed lncRNAs were identified using the Benjamini-Hochberg method. Correlation analyses of subtypes, clinicopathological factors, risk score, immune check inhibitors, and immune infiltration levels were conducted using a Pearson correlation test. Survival analysis was conducted using the Kaplan-Meier method and validated via the log-rank test. The predictive efficiencies of the ferroptosis-related lncRNA signatures for 1-, 3-, and 5-year overall survival (OS) were assessed using ROC curves. *p* < 0.05 was considered statistically significant.

## 3. Results

### 3.1. Ferroptosis-Related lncRNAs in Colon Cancer

**Figure 1:**
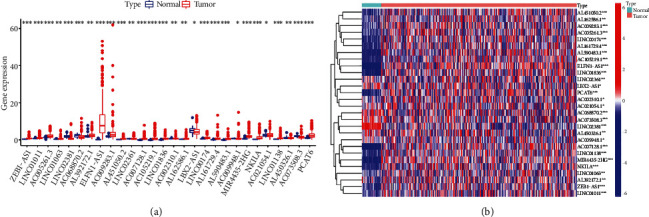
Differential expression of ferroptosis-related lncRNAs in colon cancer and adjacent normal tissues. (a) Boxplot. (b) Heatmap. ^∗^*p* < 0.05, ^∗∗^*p* < 0.01, and ^∗∗∗^*p* < 0.001. The univariate Cox regression analysis results showed that 28 ferroptosis-related lncRNAs correlated with overall survival (OS) of colon cancer patients from TCGA database.

We confirmed 1,241 differentially expressed ferroptosis-related lncRNAs of 39 normal colon tissues and 379 colon cancer tissues via coexpression analyses between ferroptosis genes and differentially expressed lncRNAs (*p* < 0.01, Pearson′s correlation coefficient > 0.3). Univariate Cox regression analyses were conducted to screen overall prognostic ferroptosis-related lncRNAs for 28 differentially expressed lncRNAs: ZEB1-AS1, LINC01011, AC005261.3, LINC01063a, LINC02381, AC068870.2, AL392172.1, ELFN1-AS1, AC009283.1, AL451050.2, LINC02361, AC007128.1, AC105219.1, LINC01836, AC002310.1, AL162586.1, LBX2-AS1, LINC00174, AL161729.4, AL590483.1, AC009948.1, MIR4435-2HG, NKILA, AC021054.1, LINC01138, AL450326.1, AC073508.3, and PCAT6. These lncRNAs were significantly related to the OS of colon cancer patients (*p* < 0.05; Table S2). Compared with normal colon tissues, four of these lncRNAs (LINC02381, LBX2-AS1, AL450326.1, and AC073508.3) were downregulated in cancer tissues, and 24 were upregulated. Figures 1(a) and 1(b) show the box plots and heatmap.

### 3.2. Consensus Clustering for Ferroptosis-Related lncRNAs Was Significantly Correlated with the Characteristics and Survival of Patients with Colon Cancer

**Figure 2:**
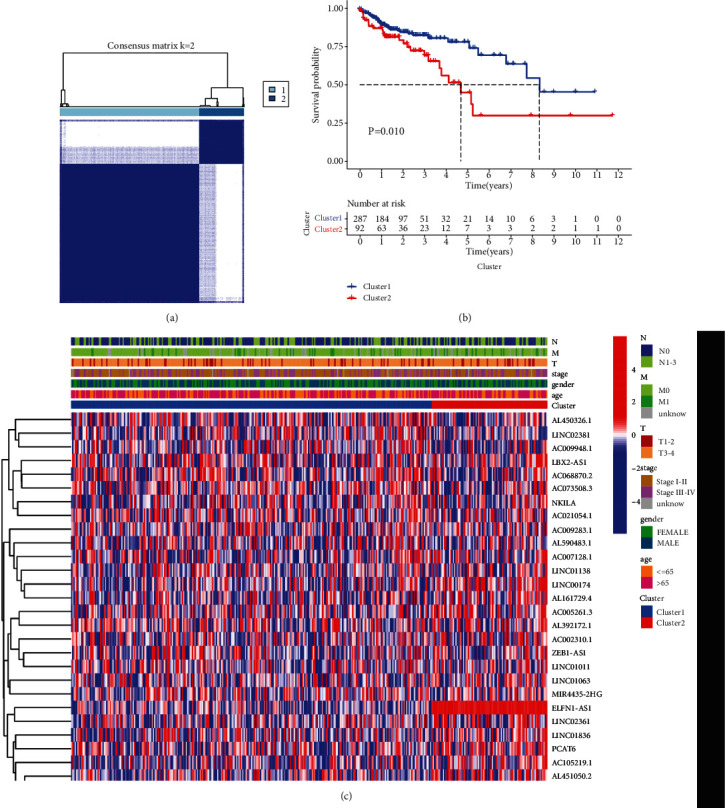
Differential clinicopathological features and overall survival of colon cancer patients in clusters 1 and 2. (a) Consensus clustering matrix for *k* = 2. (b) The Kaplan-Meier curves of overall survival (OS) for patients with colon cancer in clusters 1 and 2; *p* < 0.05. (c) Heatmap and clinicopathologic features of clusters 1 and 2.

To explore the effect of ferroptosis-related lncRNAs in progression of colon cancer, the tumor samples were divided into clusters via the ConsensusClusterPlus R package. We found that *k* = 2 showed optimal clustering stability from *k* = 2 to *k* = 9 according to the cumulative distribution function (CDF) curve of the consensus score (Figure 2(a)). The 379 patients with colon cancer were clustered into clusters 1 (*n* = 287) and 2 (*n* = 92). Supplementary Figure 1 shows the CDF curve, relative change in area under the CDF curve, and tracking plots. The OS of cluster 2 was shorter than that of cluster 1 (Figure 2(b)). The clinicopathological features did not differ between the two clusters (Table S3), indicating no heterogeneity between the two clusters (Figure 2(c)).

### 3.3. Consensus Clustering for Ferroptosis-Related lncRNAs Associated with Immune Cell Infiltration and Immune Checkpoint Inhibitors

**Figure 3:**
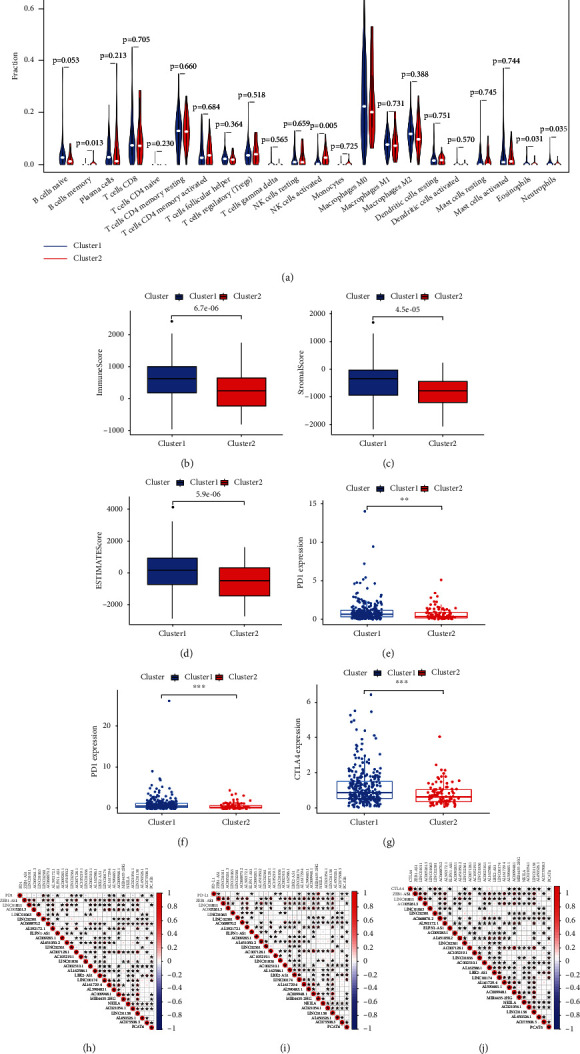
Distinct immune cell infiltration in two clusters and association of immune checkpoint inhibitors in colon cancer. (a) Infiltration levels of 22 immune cell types in clusters 1 and 2, (b) immunoscores in clusters 1 and 2, (c) stromal scores in clusters 1 and 2, (d) estimated scores in clusters 1 and 2, (e) PD1 expression levels in clusters 1 and 2, (f) PDL1 expression levels in clusters 1 and 2, (g) CTLA4 expression levels in clusters 1 and 2, (h) correlation between PD1 expression level and differential expression of ferroptosis-related lncRNAs, (i) correlation between PDL1 expression level and differential expression of ferroptosis-related lncRNAs, and (j) correlation between CTLA4 expression level and differential expression of ferroptosis-related lncRNAs, ^∗∗^*p* < 0.01 and ^∗∗∗^*p* < 0.001.

To further explore the reason for the different OS between the two clusters, we investigated the infiltration fractions of 22 immune cells (B cells naive, B cells memory, plasma cells, T cells CD8, T cells CD4 naive, T cells CD4 memory resting, T cells CD4 memory activated, T cells follicular helper, T cells regulatory (Tregs), T cells gamma delta, NK cells resting, NK cells activated, monocytes, macrophages M0, macrophages M1, macrophages M2, dendritic cells resting, dendritic cells activated, mast cells resting, mast cells activated, eosinophils, and neutrophils). Cluster 1 was more strongly correlated with eosinophils and neutrophils; however, cluster 2 had higher infiltration levels of activated memory B cells and natural killer (NK) cells (Figure 3(a)). We further analyzed the immune, stromal, and estimated scores between the two clusters and found that cluster 1 had higher immune, stromal, and estimated scores (Figures 3(b)–3(d)), demonstrating that ferroptosis-related lncRNAs regulated the tumor microenvironment to affect patients' prognoses.

Ferroptosis is reported to enhance the effect of immunotherapy by regulating immune responses [17, 18]. Thus, we explored whether the expression of ferroptosis-related lncRNAs was correlated with immune checkpoint inhibitors such as PD1, PDL1, and CTLA4. Difference analyses showed that PD1, PDL1, and CTLA4 expression levels were significantly higher in cluster 1 (Figures 3(e)–3(g)). PD1 expression was significantly positively associated with LINC02381 expression levels and significantly negatively correlated with ELFN1-AS1 expression levels (Figure 3(h)). PDL1 expression was significantly positively associated with LINC02381 and LINC02361 expression levels and significantly negatively correlated with AL590483.1 expression levels (Figure 3(i)). CTLA4 expression was significantly positively associated with LINC01011, AC005261.3, AL392172.1, AC007128.1, AC002310.1, AL162586.1, AL161729.4, AC009948.1, and LINC01138 (Figure 3(j)).

**Figure 4:**
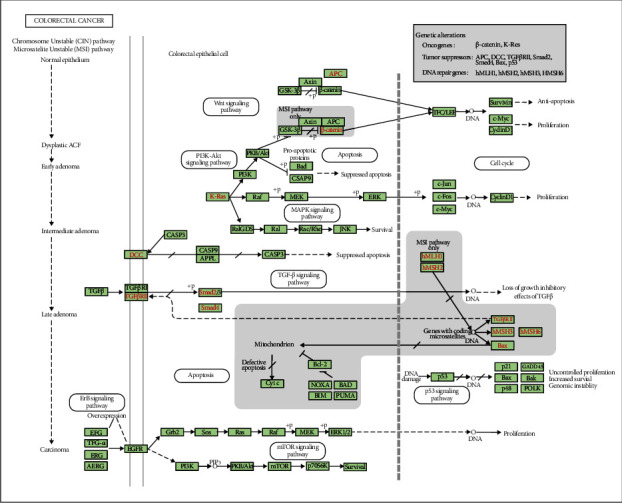
Enriched KEGG pathway for colorectal cancer.

GSEA was used to elucidate the differences in biological functions between the two clusters (Table S4). The top five pathways enriched in cluster 1 were the Kyoto Encyclopedia of Genes and Genomes (KEGG) TGF-beta signaling pathway, KEGG pathways in cancer, KEGG focal adhesion, KEGG renal cell carcinoma, and KEGG regulation of actin cytoskeleton, which are mainly related to tumorigenesis and tumor metastasis (Supplementary Figure 2 A–E). The KEGG colorectal cancer pathway (Figure 4 and Supplementary Figure 2K) was also enriched, and the Wnt, PI3K-AKT, ErbB, TGF-*β* and p53 signaling pathways were included. The top five pathways enriched in cluster 2 were KEGG RNA polymerase, KEGG ribosome, KEGG glycine serine and threonine metabolism, KEGG base excision repair, and KEGG pentose phosphate pathway, which are mainly related to tumor metabolism (Supplementary Figure 2 F–J). Thus, the ferroptosis-related lncRNAs may affect immune cell functions via cancer- and metabolism-related pathways and may affect patient prognoses.

### 3.4. Construction and Validation of Prognostic Signatures for Ferroptosis-Related lncRNAs

The 379 patients were randomly divided into the training (191 patients) and testing (188 patients) cohorts. To precisely predict the clinical outcomes of colon cancer patients, we performed the LASSO regression analysis based on the expression values of the ferroptosis-related lncRNAs, which were screened via the univariate Cox regression analyses (Supplementary Figure 3). We identified 15 lncRNAs: ZEB1-AS1, LINC01011, AC005261.3, LINC01063, LINC02381, ELFN1-AS1, AC009283.1, LINC02361, AC105219.1, AC002310.1, AL590483.1, MIR4435-2HG, NKILA, AC021054.1, and AL450326.1. We then constructed and validated a 15 ferroptosis-related lncRNA signature to predict patient prognoses using the training and testing cohorts and the risk score equation = 0.0019 × Exp (ZEB1 − AS1) + 0.1752 × Exp (LINC01011) + 0.2194 × Exp(AC005261.3) + 0.3713 × Exp(LINC01063) + 0.0683 × Exp(LINC02381) + 0.0276 × Exp(ELFN1 − AS1) + 0.0450 × Exp (AC009283.1) + 0.3155 × Exp(LINC02361) + 0.1286 × Exp(AC105219.1) + 0.1528 × Exp(AC002310.1) + −1.1310 × Exp(AL590483.1) + 0.1252 × Exp(MIR4435 − 2HG) + 0.2576 × Exp(NKILA) + 0.1841 × Exp(AC021054.1) + 0.2207 × Exp(AL450326.1).

**Figure 5:**
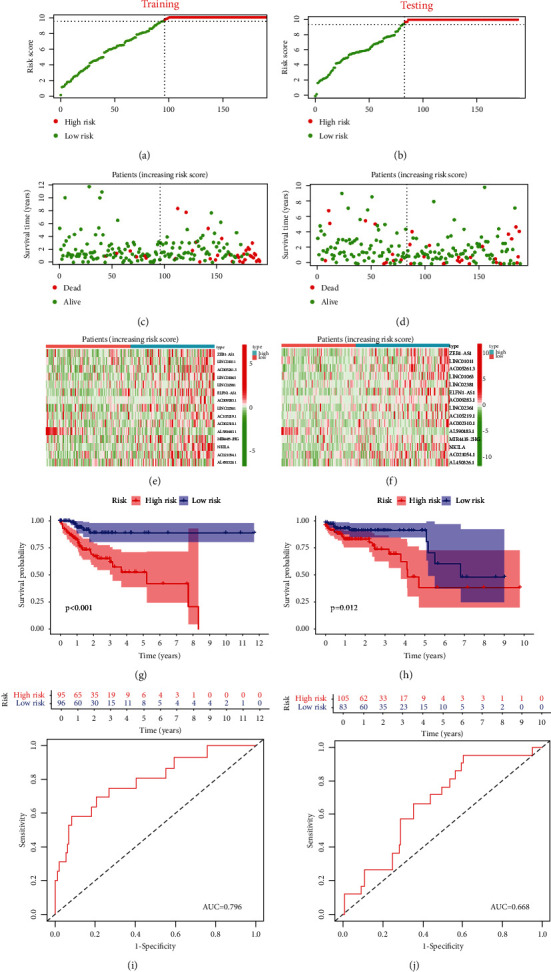
Construction and validation of prognostic signature of 15 ferroptosis-related lncRNAs in the training and testing cohorts. (a, b) Risk score distribution. (c, d) OS status. (e, f) Heatmaps. (g, h) The Kaplan-Meier curves for OS. (i, j) ROC curves.

Patients in the training and testing cohorts were divided into the high- or low-risk group according to their median risk score. The risk score distribution, survival overview, and gene expression heatmaps of the 15 ferroptosis-related lncRNA-based signatures in the training (Figures 5(a), 5(c), and 5(e)) and testing (Figures 5(b), 5(d), and 5(f)) cohorts are shown. The survival analysis illustrated that the high-risk group had a significantly worse OS compared with that of the low-risk group in both cohorts (Figures 5(g) and 5(h)). The areas under the time-dependent ROC curve of the training and testing cohorts were 0.796 and 0.668, suggesting that the risk scores calculated based on the 15 ferroptosis-related lncRNA signatures had a good prediction performance.

### 3.5. Prognostic Risk Scores Correlated with Clinicopathological Characteristics

**Figure 6:**
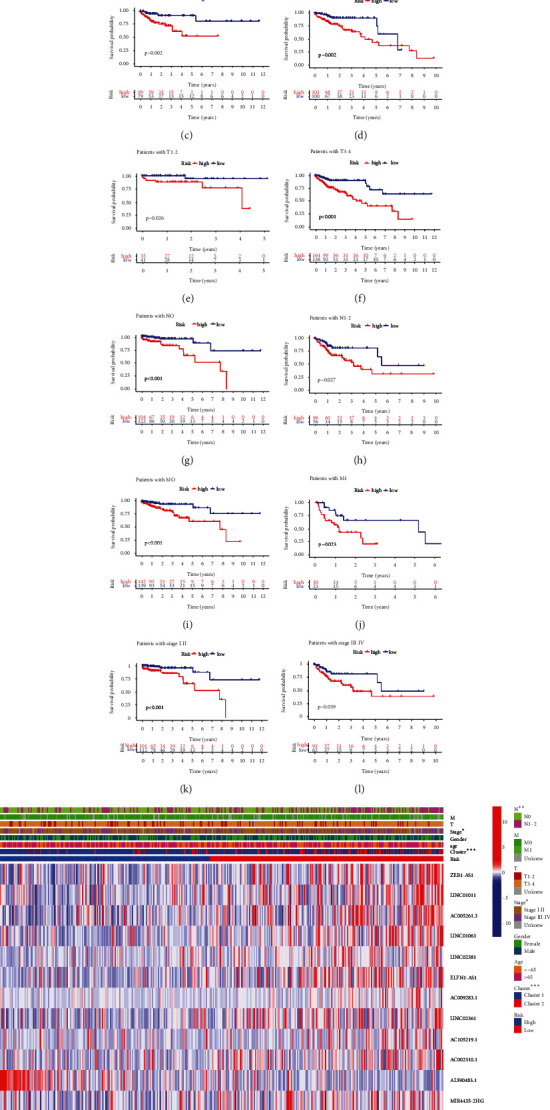
The Kaplan-Meier survival subgroup analysis for the prognostic signature of 15 ferroptosis-related lncRNAs stratified by clinical characteristics. (a, b) patients aged > 65 years and ≤ 65 years. (c, d) Female and male. (e, f) T1-2 and T3-4. (g, h) N0 and N1-2. (i, j) M0 and M1. (k, l) Stages I–II and III–IV. (m) Heatmap for differential clinicopathological features of high- and low-risk scores.

We evaluated the connections between risk score, clinicopathological features of 379 colon cancer patients, and expression levels of 15 ferroptosis-related lncRNAs. The OS of the high-risk group was significantly shorter than that of the low-risk group in patients aged both > 65 and ≤ 65 years; in both sexes; in T1-2, T3-4, N0, N1-2, M0, and M1 classifications; and in stages I–II and III–IV (Figures 6(a)–6(l)). The heatmap demonstrated that high-risk patients were significantly correlated with N classification, stage, and cluster (Figure 6(m)).

**Figure 7:**
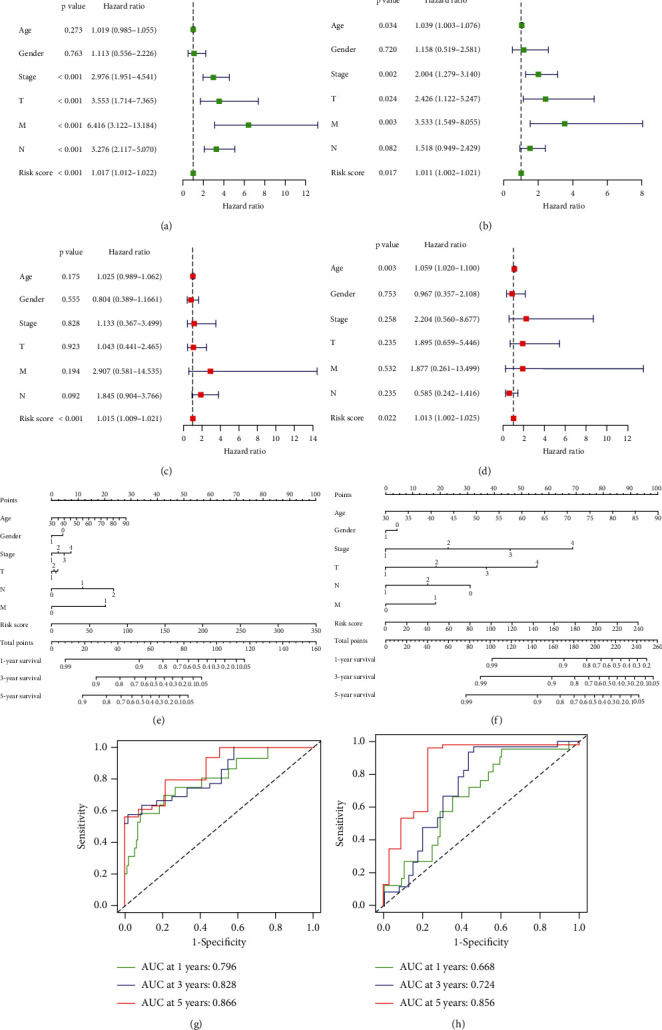
Independent analysis and construction of nomogram and performance assessment. (a, b) The univariate Cox regression analysis of prognostic factors. (c, d) The multivariate Cox regression analysis of prognostic factors. (e, f) Nomogram based on clinical factors and risk score. (g, h) Nomogram of AUC values for 1-, 3-, and 5-year survival rates.

Univariate and multivariate Cox regression analyses were performed in both cohorts to explore whether the risk score independently predicted the prognoses of patients with colon cancer. The univariate Cox regression showed that stage, TNM classification, and risk score were significantly associated with OS in the training cohort (Figure 7(a)). The multivariate Cox regression confirmed that risk score was an independent prognostic indicator in the training cohort (Figure 7(c)). In the testing cohort, the univariate Cox analysis showed that age, stage, TM classification, and risk score were significantly associated with OS (Figure 7(b)). The multivariate Cox regression showed that age and risk score were independent prognostic indicators (Figure 7(d)). The results showed that risk score was an independent prognostic factor for colon cancer patients.

Prognostic nomograms of both cohorts incorporating clinicopathological characteristics and the prognostic signature of 15 ferroptosis-related lncRNAs were established to provide a quantitative and visual method for predicting the 1-, 3-, and 5-year OS probabilities of colon cancer patients (Figures 7(e) and 7(f)). The area under the ROC curve (AUC) values of the training cohort for 1-, 3-, and 5-year OS were 0.796, 0.828, and 0.866, respectively (Figure 7(g)). In the testing cohort, the AUC values for 1-, 3-, and 5-year OS were 0.668, 0.724, and 0.856, respectively (Figure 7(h)). From these findings, we concluded that the prognostic signature of the 15 ferroptosis-related lncRNAs could independently predict the prognosis and may be applied to clinically manage colon cancer patients.

### 3.6. Estimation of Immune Cell Infiltration and Chemotherapeutic Correlation of the Ferroptosis-Related lncRNA Signature

**Figure 8:**
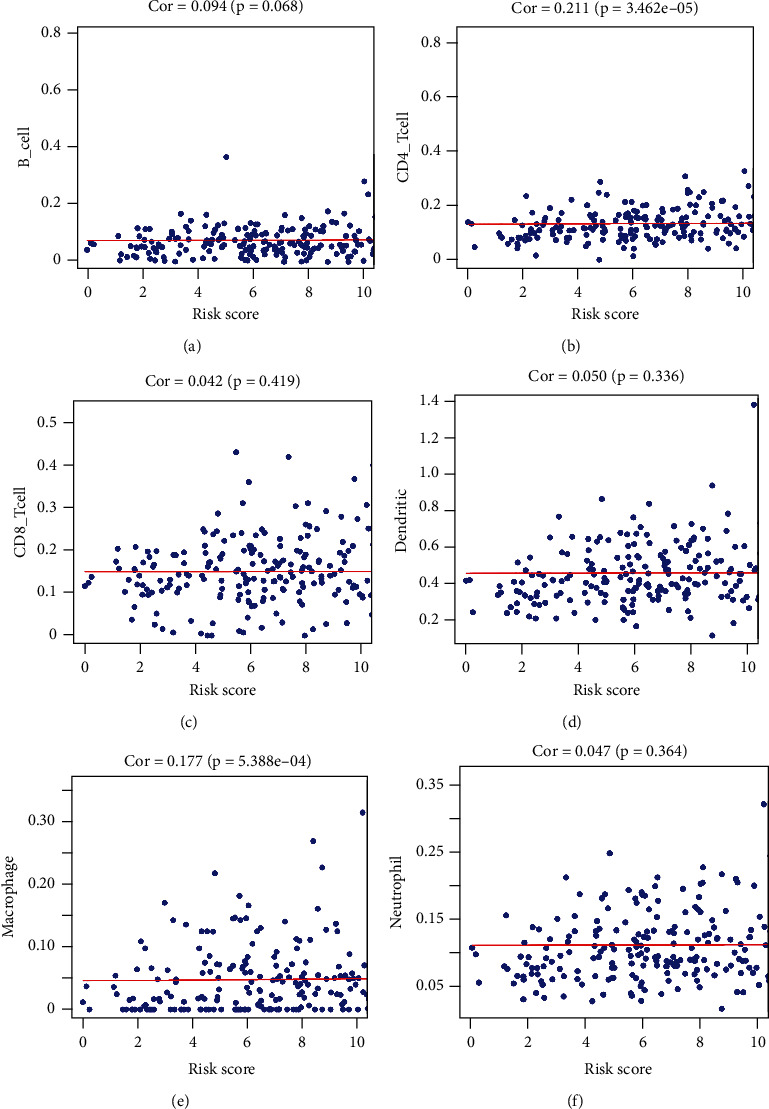
Relationships between risk score and infiltration abundances of six immune cell types. (a) B cells, (b) CD4 T cells, (c) CD8 T cells, (d) dendritic cells, (e) macrophages, and (f) neutrophils. The results showed that CD4 T cell was significantly positively correlated with risk score (*p* < 0.05). B cell, CD8 T cell, dendritic cell, macrophage, and neutrophil are not correlated with risk score (*p* > 0.05).

Because ferroptosis is related to the immune microenvironment, we investigated the relationship between the risk score and immune cell infiltration to estimate the effect of the prognostic signature of the 15 ferroptosis-related lncRNAs on the colon cancer immune microenvironment. CD4 T cell was significantly positively correlated with risk score (Figure 8(b)). B cell, CD8 T cell, dendritic cell, macrophage, and neutrophil were not correlated with risk score (Figures 8(a) and 8(c)–8(f)).

**Figure 9:**
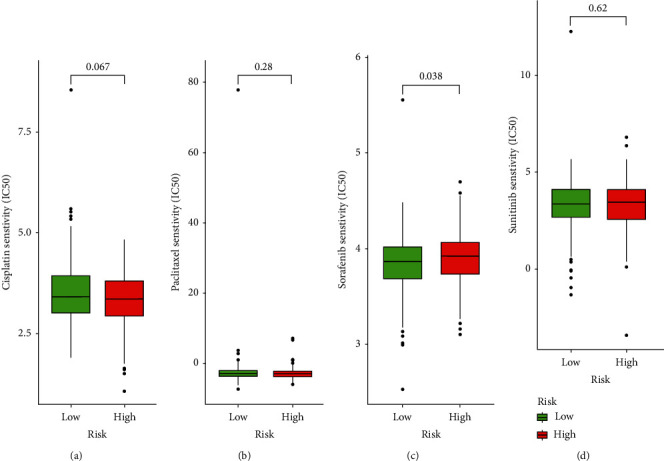
Correlation between risk scores and chemotherapeutic drugs. (a) Cisplatin, (b) paclitaxel, (c) sorafenib, and (d) sunitinib. The results showed that sorafenib was significantly positively correlated with risk score (*p* < 0.05). Cisplatin, paclitaxel, and sunitinib are not correlated with risk score (*p* > 0.05).

To use the signature to help clinicians determine the best treatment, we attempted to identify the association between risk score and the efficacies of common chemotherapeutics in treating colon cancer. For sorafenib, a high-risk score was associated with a higher chemotherapeutic IC50, demonstrating that the signature is a potential predictor of chemosensitivity (Figure 9(c)). However, cisplatin, paclitaxel, and sunitinib showed no correlation with scores (Figures 9(a), 9(b), and 9(d)).

## 4. Discussion

Owing to the absence of easily observable symptoms, colon cancer is often discovered at a late stage during a patient's clinical course, and most patients with colon cancer succumb to the disease owing to distant metastasis [19]. Tumor progression is dependent on the tumor microenvironment [20], as well as on the characteristics of the tumor cells, and inflammation in the tumor microenvironment [21–23]. Conventional systems for predicting prognoses, such as the American Joint Committee on cancer TNM and Duke staging systems, cannot accurately predict the prognoses of cancer patients. Further research has shown that the molecular subtypes of colon cancer can help define prognostic factors, predict patient survival, and indicate treatment. Ferroptosis is a newly defined mode of programmed oxidative cell death, but its specific role and effect on the prognoses of colon cancer patients remain unclear. The current study is the first to divide ferroptosis-related lncRNAs into different subgroups, construct a prognostic signature, and systematically investigate the correlation between the tumor microenvironment, immune cell infiltration, immune check inhibitors, and ferroptosis-related lncRNAs to indicate treatment.

Our study identified and validated two ferroptosis-related lncRNA subtypes in colon cancer. Cluster 2 had a worse OS than did cluster 1. The tumor microenvironment plays an important regulatory role in promoting tumor growth, and its heterogeneity affects patients' prognoses and therapeutic responses [24]. It had been reported that spatial and temporal heterogeneity for lymphocyte infiltration in advanced urothelial cancer and that CD3^+^, CD8^+^, and FoxP3^+^ cell densities decreased during treatment with platinum-based chemotherapy [25]. Thus, we explored the immune cell infiltration landscape and tumor microenvironment of the two subtypes. Cluster 1 had more eosinophils and neutrophils and fewer activated memory B cells and NK cells than did cluster 2. Differences in the tumor microenvironments were also explored, and cluster 1 had higher immune, stromal, and estimated scores. Differences in PD1, PDL1, and CTLA4 expression levels were significantly higher in cluster 1. These results indicated that cluster 1 displayed a superior response to immunotherapy. A previous report of triple-negative breast cancer demonstrated that a marked reduction in the percentage of CD8^+^ T lymphocytes and a significant increase in the frequencies of CD4^+^ T lymphocytes and CD4^+^ and CD8^+^ T lymphocytes expressing PD1 and CD39 were evident in tumor tissue in comparison with the normal breast tissue [26]. It was also found that an increased percentage of activated CD4^+^CD25^+^Foxp3^−^ and CD8^+^CD25^+^ T cells reduced tumor progression during colorectal cancer development in vivo [27]. We further showed that risk score was positively correlated with the CD4^+^ T cell infiltration levels and was not correlated with B cell, CD8 T cell, dendritic cell, macrophage, and neutrophil. To study the clinical application value, we conducted chemosensitivity analyses, which showed that low-risk-score patients were more sensitive to sorafenib, but not sensitive to cisplatin, paclitaxel, and sunitinib. These findings suggested that ferroptosis-related lncRNAs are partially involved in regulating the tumor microenvironment and may facilitate personalized treatment for patients with colon cancer.

GSEA elucidated the specific mechanisms of the two subtypes. The ferroptosis-related lncRNAs were mainly involved in tumorigenesis, metastasis, and metabolism-related pathways. The KEGG colorectal cancer pathway was enriched, and the Wnt, PI3K-AKT, ErbB, TGF-*β*, and p53 signaling pathways were involved. Previous studies confirmed that some of these pathways were involved in regulating ferroptosis in different cancers. A study highlighted that *Andrographis* could activate ferroptosis and suppress the *β*-catenin/Wnt signaling pathways to mediate chemosensitization in colorectal cancer [28]. Hyperactive mutation of PI3K-AKT-mTOR signaling protects cancer cells from oxidative stress and ferroptotic death through SREBP1/SCD1-mediated lipogenesis [29]. XCT expression was repressed by TGF-*β*1 by activating Smad3 and enhancing lipid peroxidation in hepatocellular carcinoma cells [30]. Mutant p53 sensitized tumor cells to ferroptosis [31]. These results provide critical references for individualized treatment of patients with colon cancer.

Finally, we constructed and validated a ferroptosis-related lncRNA prognostic signature in two independent cohorts. Some lncRNAs that constituted the signature were related to colon cancer pathogeneses and progression. ELFN1-AS1 drives colon cancer cells to proliferate and invade by adjusting the miR-191-5p/SATB1 axis [32]. lncRNA MIR4435-2HG-mediated cisplatin resistance occurs via the Nrf2/HO-1 pathway in colon cancer [33]. No reports have been published on other lncRNAs in colon cancer; thus, our future studies will focus on those lncRNAs. Survival and clinicopathological analyses demonstrated that the signature performed favorably in predicting the prognosis and was an independent prognostic factor for colon cancer. Nomograms provided a quantitative and visual method for predicting the 1-, 3-, and 5-year OS probabilities of colon cancer patients. ROC curves indicated that the ferroptosis-related lncRNA prognostic signature was highly accurate and reliable.

The present study differs from previous studies [34, 35] regarding the ferroptosis-related prognostic signature of colon cancer and has some advantages. First, consensus clustering for ferroptosis-related lncRNAs was used in colon cancer. Second, because colon cancer is a typical microsatellite-instable tumor, its correlation with ferroptosis requires further study. This study comprehensively analyzed the relationship between the ferroptosis-related lncRNA prognostic signature and the tumor microenvironment as well as immune cell infiltration, providing a new perspective on the predictive function of ferroptosis-related lncRNA signatures in immunotherapy. Third, this study explored the correlation between the ferroptosis-related lncRNA signature and immune checkpoint inhibitor expression and chemosensitivity. This may help clinicians to more accurately choose the best clinical policy and therapy for patients with colon cancer.

Our study had several limitations. First, the main datasets in our study were obtained from TCGA, and other datasets as well as our own data should be obtained to reduce selection bias. Additionally, some crucial clinicopathological parameters, such as CEA, MSI, dMMR, KRAS, NRAS, and BRAF mutation statuses, were not obtained in the nomogram; thus, the function of this ferroptosis-related lncRNA signature must be validated in clinical research.

## 5. Conclusion

In summary, we systematically evaluated the prognostic value, the role in the tumor microenvironment and immune cell infiltration, the potential regulatory mechanisms of ferroptosis-related lncRNAs, and the correlation between immune checkpoint inhibitors and chemosensitivity with colon cancer. The identified signature of 15 ferroptosis-related lncRNAs accurately predicted the prognoses of patients with colon cancer and may help determine individual therapeutic strategies and expand on insights for advancing therapeutic approaches.

## Data Availability

The datasets analyzed in this study can be found in public online databases. Raw counts of RNA-seq transcriptome data and corresponding clinical data for colon cancer were extracted from TCGA (https://portal.gdc.cancer.gov). The ferroptosis-related genes were extracted from FerrDb (http://www.zhounan.org/ferrdb). Infiltration data for 22 immune cells were downloaded from the TIMER (http://timer.cistrome.org) and CIBERSORT (https://cibersort.stanford.edu/) databases.

## Supplementary Materials

Supplementary Materials Table S1: ferroptosis-related genes downloaded from FerrDb. Table S2: the univariate Cox regression analysis of ferroptosis-related genes. Table S3: characteristics of the two clusters of COAD patients. Table S4: detailed enrichment results of the GSEA in clusters 1 and 2. Figure S1: consensus clustering of the tumorous cohort from TCGA based on differentially expressed ferroptosis-related lncRNAs. Figure S2: distinct pathways enriched in clusters 1 and 2. Figure S3: Fifteen ferroptosis-related lncRNAs were identified via the LASSO regression analysis.
