# Supplementary material for: Comprehensive Analysis of Immune Infiltrates of Ferroptosis-Related Long Noncoding RNA and Prediction of Colon Cancer Patient Prognoses
Source: J Immunol Res. 2022 Feb 27;2022:9480628. doi: 10.1155/2022/9480628 (PMC8898846; doi:10.1155/2022/9480628)
Supplement: Supplementary Materials — Table S1: ferroptosis-related genes downloaded from FerrDb. Table S2: the univariate Cox regression analysis of ferroptosis-related genes. Table S3: characteristics of the two clusters of COAD patients. Table S4: detailed enrichment results of the GSEA in clusters 1 and 2. Figure S1: consensus clustering of the tumorous cohort from TCGA based on differentially expressed ferroptosis-related lncRNAs. Figure S2: distinct pathways enriched in clusters 1 and 2. Figure S3: Fifteen ferroptosis-related lncRNAs were identified via the LASSO regression analysis. [file 9480628.f1.zip › 9480628.f1/Supplementary Table 1 (1).pdf]

ABCC1	BECN1	EMC2	HSD17B11	MIR30B	PRDX1	SQSTM1
ACO1	BID	ENPP2	HSF1	MIR4715	PRDX6	SRC
ACSF2	BLOC1S5-TXNDC5	EPAS1	HSPA5	MIR6852	PRKAA1	SRXN1
ACSL3	BNIP3	FADS2	HSPB1	MIR9-1	PRKAA2	STAT3
ACSL4	BRD4	FANCD2	IDH1	MIR9-2	PROM2	STEAP3
ACVR1B	CA9	FBXW7	IFNG	MIR9-3	PSAT1	STMN1
AGPAT3	CAPG	Fer1HCH	IL33	MT1G	PTGS2	TAZ
AIFM2	CARS1	FH	IL6	MT3	RB1	TF
AKR1C1	CAV1	FLT3	IREB2	MTDH	RELA	TFAP2C
AKR1C2	CBS	FTH1	ISCU	MTOR	RGS4	TFR2
AKR1C3	CD44	FTL	JDP2	MUC1	RIPK1	TFRC
ALB	CDKN1A	FTMT	JUN	MYB	RPL8	TGFBR1
ALOX12	CDKN2A	G6PD	KEAP1	NCF2	RRM2	TLR4
ALOX12B	CDO1	G6PDX	KIM-1	NCOA4	SAT1	TMBIM4
ALOX15	CEBPG	GABARAPL1	KLHL24	NF2	SCD	TNFAIP3
ALOX15B	CHAC1	GABARAPL2	KRAS	NFE2L2	SCP2	TP53
ALOX5	CHMP5	GABPB1	LAMP2	NFS1	SELENOS	TP63
ALOXE3	CHMP6	GCH1	LINC00336	NGB	SESN2	TRIB3
ANGPTL7	CISD1	GCLC	LINC00472	NNMT	SETD1B	TSC22D3
ANO6	CISD2	GDF15	LOC284561	NOS2	SIRT1	TUBE1
ARNTL	CS	GLS2	LOC390705	NOX1	SLC1A4	TXNIP
ARRDC3	CXCL2	GLUT13	LONP1	NOX3	SLC1A5	TXNRD1
ASNS	CYBB	GOT1	LPCAT3	NOX4	SLC2A1	UBC
ATF3	DDIT3	GPT2	LPIN1	NOX5	SLC2A12	ULK1
ATF4	DDIT4	GPX2	LURAP1L	NQO1	SLC2A14	ULK2
ATG13	DNAJB6	GPX4	MAFG	NRAS	SLC2A3	VDAC2
ATG16L1	DPP4	HAMP	MAP1LC3A	OTUB1	SLC2A6	VEGFA
ATG3	DRD4	HBA1	MAP3K5	OXSR1	SLC2A8	VLDLR
ATG4D	DRD5	HELLS	MAPK1	PANX1	SLC38A1	WIPI1
ATG5	DUOX1	HERPUD1	MAPK14	PCK2	SLC3A2	WIPI2
ATG7	DUOX2	HIC1	MAPK3	PEBP1	SLC40A1	XBP1
ATM	DUSP1	HIF1A	MAPK8	PGD	SLC7A11	YWHAE
ATP5MC3	EGFR	HILPDA	MAPK9	PHKG2	SLC7A5	YY1AP1
ATP6V1G2	EGLN2	HMGB1	MIOX	PIK3CA	SNORA16A	ZEB1
AURKA	EIF2AK4	HMOX1	MIR137	PLIN2	SNX4	ZFP36
BACH1	EIF2S1	HNF4A	MIR17	PLIN4	SOCS1	ZFP69B
BAP1	ELAVL1	HRAS	MIR212	PML	SP1	ZNF419
