# Supplementary material for: Comprehensive Analysis of Immune Infiltrates of Ferroptosis-Related Long Noncoding RNA and Prediction of Colon Cancer Patient Prognoses
Source: J Immunol Res. 2022 Feb 27;2022:9480628. doi: 10.1155/2022/9480628 (PMC8898846; doi:10.1155/2022/9480628)
Supplement: Supplementary Materials — Table S1: ferroptosis-related genes downloaded from FerrDb. Table S2: the univariate Cox regression analysis of ferroptosis-related genes. Table S3: characteristics of the two clusters of COAD patients. Table S4: detailed enrichment results of the GSEA in clusters 1 and 2. Figure S1: consensus clustering of the tumorous cohort from TCGA based on differentially expressed ferroptosis-related lncRNAs. Figure S2: distinct pathways enriched in clusters 1 and 2. Figure S3: Fifteen ferroptosis-related lncRNAs were identified via the LASSO regression analysis. [file 9480628.f1.zip › 9480628.f1/Supplementary Table 2 (1).pdf]

gene	KM	SE	HR	HR.95L	HR.95H	pvalue
ZEB1-AS1	0.0034263	0.1951989	2.4191584	1.6501017	3.5466467	6.02E-06
LINC01011	0.0086154	0.1885965	1.6618844	1.1483311	2.4051073	0.0070743
AC005261.3	0.0205063	0.0931651	1.2022905	1.0016297	1.4431506	0.0479917
LINC01063	0.0329139	0.1474595	1.6821504	1.2599319	2.2458595	0.0004205
LINC02381	0.007924	0.0814575	1.3068897	1.1140437	1.5331183	0.0010171
AC068870.2	0.0175597	0.0631554	1.2527471	1.1068924	1.4178209	0.0003597
AL392172.1	0.0384032	0.0871739	1.1940675	1.0065291	1.4165483	0.0418893
ELFN1-AS1	0.0109066	0.0104878	1.0254207	1.0045576	1.0467172	0.0166869
AC009283.1	0.0361493	0.0175846	1.0535992	1.0179054	1.0905446	0.0029858
AL451050.2	0.0430549	0.1912633	1.5022445	1.0326116	2.185467	0.0333579
LINC02361	0.0108136	0.1885089	1.6492679	1.1398092	2.3864384	0.0079508
AC007128.1	0.013517	0.1919891	1.5397941	1.0569178	2.243283	0.0245572
AC105219.1	0.0438772	0.0652773	1.1423577	1.0051664	1.2982737	0.0414595
LINC01836	0.0029615	0.1221902	1.3663518	1.0753605	1.7360851	0.0106317
AC002310.1	0.0111605	0.1393695	1.9038175	1.448751	2.5018247	3.84E-06
AL162586.1	0.0034326	0.1271214	1.494545	1.1649386	1.9174099	0.0015727
LBX2-AS1	0.0033073	0.0473567	1.1077821	1.0095881	1.2155265	0.0306594
LINC00174	0.0429056	0.1320271	1.3473456	1.0401534	1.7452621	0.0239365
AL161729.4	0.0152543	0.120332	1.338834	1.0575476	1.6949368	0.0153105
AL590483.1	0.0330238	0.2518495	0.5821355	0.3553444	0.9536714	0.0316887
AC009948.1	0.044301	0.1388774	1.3263465	1.0102861	1.741284	0.0419867
MIR4435-2HG	0.011655	0.1209462	1.3238828	1.0444795	1.6780279	0.0203527
NKILA	0.0304782	0.0642576	1.3101027	1.1550724	1.4859408	2.63E-05
AC021054.1	0.0481077	0.0743741	1.1617585	1.004173	1.344074	0.0438046
LINC01138	0.0207535	0.1917961	1.84779	1.2688067	2.6909754	0.0013683
AL450326.1	0.0152385	0.2042992	1.6161398	1.082877	2.412008	0.0187889
AC073508.3	0.0430079	0.1063854	1.3784778	1.1190367	1.6980684	0.0025517
PCAT6	0.0434752	0.0590962	1.2318182	1.0970941	1.3830867	0.0004187
