# Supplementary material for: Comprehensive Analysis of Immune Infiltrates of Ferroptosis-Related Long Noncoding RNA and Prediction of Colon Cancer Patient Prognoses
Source: J Immunol Res. 2022 Feb 27;2022:9480628. doi: 10.1155/2022/9480628 (PMC8898846; doi:10.1155/2022/9480628)
Supplement: Supplementary Materials — Table S1: ferroptosis-related genes downloaded from FerrDb. Table S2: the univariate Cox regression analysis of ferroptosis-related genes. Table S3: characteristics of the two clusters of COAD patients. Table S4: detailed enrichment results of the GSEA in clusters 1 and 2. Figure S1: consensus clustering of the tumorous cohort from TCGA based on differentially expressed ferroptosis-related lncRNAs. Figure S2: distinct pathways enriched in clusters 1 and 2. Figure S3: Fifteen ferroptosis-related lncRNAs were identified via the LASSO regression analysis. [file 9480628.f1.zip › 9480628.f1/Supplementary Table 3 (1).pdf]

**Characteristics**	**Entire cohort (n=379)**	**Cluster1 (n=287)**	**Cluster2 (n=92)**	***P* value**
**Age, years**				
<=65	157	121	36	0.608
> 65	222	166	56	
**Sex**				
Female	178	133	45	0.667
Male	201	154	47	
**pT stage**				
T1-2	76	56	20	0.770
T3-4	302	230	72	
Unknow	1	1	0	
**pN stage**				
N0	227	175	52	0.448
N1-2	152	112	40	
**pM stage**				
M0	281	219	62	0.166
M1	53	35	18	
Unknow	45	33	12	
**TNM stage**				
Stage I-II	213	165	48	0.669
Stage III-IV	155	114	41	
Unknow	11	8	3	
