# Supplementary material for: Comprehensive Analysis of Immune Infiltrates of Ferroptosis-Related Long Noncoding RNA and Prediction of Colon Cancer Patient Prognoses
Source: J Immunol Res. 2022 Feb 27;2022:9480628. doi: 10.1155/2022/9480628 (PMC8898846; doi:10.1155/2022/9480628)
Supplement: Supplementary Materials — Table S1: ferroptosis-related genes downloaded from FerrDb. Table S2: the univariate Cox regression analysis of ferroptosis-related genes. Table S3: characteristics of the two clusters of COAD patients. Table S4: detailed enrichment results of the GSEA in clusters 1 and 2. Figure S1: consensus clustering of the tumorous cohort from TCGA based on differentially expressed ferroptosis-related lncRNAs. Figure S2: distinct pathways enriched in clusters 1 and 2. Figure S3: Fifteen ferroptosis-related lncRNAs were identified via the LASSO regression analysis. [file 9480628.f1.zip › 9480628.f1/Supplementary Table 4.pdf]

**Gene sets enriched in phenotype C1 (287 samples)**

| NAME | SIZE | ES | NES | NOM p-val | FDR q-val | FWER p-val | RANK AT MAX | LEADING EDGE |
|---|---|---|---|---|---|---|---|---|
| KEGG_TGF_BETA_SIGNALING_PATHWAY | 86 | -0.65 | -2.28 | 0 | 0 | 0 | 8977 | tags=59%, list=16%, signal=71% |
| KEGG_PATHWAYS_IN_CANCER | 325 | -0.6 | -2.27 | 0 | 0 | 0 | 7717 | tags=49%, list=14%, signal=57% |
| KEGG_FOCAL_ADHESION | 199 | -0.69 | -2.25 | 0 | 0.001 | 0.001 | 7167 | tags=61%, list=13%, signal=70% |
| KEGG_RENAL_CELL_CARCINOMA | 70 | -0.66 | -2.24 | 0 | 0 | 0.001 | 7041 | tags=59%, list=13%, signal=67% |
| KEGG_REGULATION_OF_ACTIN_CYTOSKELETON | 213 | -0.61 | -2.22 | 0 | 0 | 0.001 | 6787 | tags=50%, list=12%, signal=57% |
| KEGG_FC_EPSILON_RI_SIGNALING_PATHWAY | 79 | -0.62 | -2.21 | 0 | 0 | 0.001 | 4660 | tags=44%, list=8%, signal=48% |
| KEGG_CHEMOKINE_SIGNALING_PATHWAY | 188 | -0.65 | -2.2 | 0 | 0.001 | 0.003 | 7768 | tags=54%, list=14%, signal=63% |
| KEGG_JAK_STAT_SIGNALING_PATHWAY | 155 | -0.61 | -2.19 | 0 | 0.001 | 0.003 | 8370 | tags=46%, list=15%, signal=55% |
| KEGG_CYTOKINE_CYTOKINE_RECEPTOR_INTERACTION | 264 | -0.65 | -2.18 | 0 | 0.001 | 0.003 | 8397 | tags=51%, list=15%, signal=60% |
| KEGG_T_CELL_RECEPTOR_SIGNALING_PATHWAY | 108 | -0.65 | -2.18 | 0 | 0.001 | 0.003 | 8547 | tags=59%, list=15%, signal=70% |
| KEGG_LEUKOCYTE_TRANSENDOTHELIAL_MIGRATION | 116 | -0.65 | -2.18 | 0 | 0.001 | 0.005 | 5441 | tags=52%, list=10%, signal=57% |
| KEGG_HEMATOPOIETIC_CELL_LINEAGE | 85 | -0.74 | -2.17 | 0 | 0.001 | 0.005 | 8321 | tags=68%, list=15%, signal=80% |
| KEGG_NATURAL_KILLER_CELL_MEDIATED_CYTOTOXICITY | 132 | -0.63 | -2.15 | 0 | 0.001 | 0.008 | 8547 | tags=49%, list=15%, signal=58% |
| KEGG_FC_GAMMA_R_MEDIATED_PHAGOCYTOSIS | 96 | -0.62 | -2.15 | 0 | 0.001 | 0.008 | 7563 | tags=55%, list=14%, signal=64% |
| KEGG_B_CELL_RECEPTOR_SIGNALING_PATHWAY | 75 | -0.66 | -2.14 | 0 | 0.001 | 0.009 | 7280 | tags=57%, list=13%, signal=66% |
| KEGG_TOLL_LIKE_RECEPTOR_SIGNALING_PATHWAY | 102 | -0.63 | -2.13 | 0 | 0.001 | 0.009 | 7398 | tags=53%, list=13%, signal=61% |
| KEGG_GAP_JUNCTION | 90 | -0.61 | -2.12 | 0 | 0.001 | 0.009 | 4293 | tags=39%, list=8%, signal=42% |
| KEGG_ADHERENS_JUNCTION | 73 | -0.64 | -2.1 | 0 | 0.001 | 0.012 | 7230 | tags=52%, list=13%, signal=60% |
| KEGG_PANCREATIC_CANCER | 70 | -0.61 | -2.09 | 0 | 0.001 | 0.016 | 7280 | tags=49%, list=13%, signal=56% |
| KEGG_PROSTATE_CANCER | 89 | -0.59 | -2.08 | 0.002 | 0.001 | 0.016 | 4712 | tags=43%, list=9%, signal=47% |
| KEGG_ECM_RECEPTOR_INTERACTION | 84 | -0.75 | -2.08 | 0.004 | 0.001 | 0.019 | 7821 | tags=70%, list=14%, signal=82% |
| KEGG_COMPLEMENT_AND_COAGULATION_CASCADES | 69 | -0.68 | -2.08 | 0.002 | 0.001 | 0.019 | 9040 | tags=57%, list=16%, signal=67% |
| KEGG_MELANOMA | 71 | -0.6 | -2.07 | 0 | 0.001 | 0.019 | 7230 | tags=49%, list=13%, signal=57% |
| KEGG_CELL_ADHESION_MOLECULES_CAMS | 131 | -0.68 | -2.06 | 0 | 0.001 | 0.022 | 9820 | tags=65%, list=18%, signal=79% |
| KEGG_NOD_LIKE_RECEPTOR_SIGNALING_PATHWAY | 62 | -0.64 | -2.05 | 0 | 0.002 | 0.025 | 7451 | tags=55%, list=13%, signal=63% |
| KEGG_ALDOSTERONE_REGULATED_SODIUM_REABSORPTION | 42 | -0.64 | -2.03 | 0 | 0.002 | 0.032 | 6475 | tags=52%, list=12%, signal=59% |
| KEGG_COLORECTAL_CANCER | 62 | -0.58 | -2.02 | 0 | 0.003 | 0.038 | 3943 | tags=42%, list=7%, signal=45% |
| KEGG_GLIOMA | 65 | -0.59 | -2.02 | 0 | 0.003 | 0.038 | 5374 | tags=48%, list=10%, signal=53% |
| KEGG_VASCULAR_SMOOTH_MUSCLE_CONTRACTION | 114 | -0.58 | -2.01 | 0 | 0.003 | 0.041 | 6876 | tags=41%, list=12%, signal=47% |
| KEGG_ACUTE_MYELOID_LEUKEMIA | 57 | -0.6 | -2 | 0.002 | 0.003 | 0.045 | 8358 | tags=54%, list=15%, signal=64% |
| KEGG_SMALL_CELL_LUNG_CANCER | 84 | -0.58 | -1.99 | 0 | 0.003 | 0.046 | 10165 | tags=58%, list=18%, signal=71% |
| KEGG_ENDOCYTOSIS | 181 | -0.51 | -1.99 | 0 | 0.003 | 0.05 | 8598 | tags=47%, list=16%, signal=55% |
| KEGG_MAPK_SIGNALING_PATHWAY | 267 | -0.52 | -1.98 | 0 | 0.003 | 0.051 | 9269 | tags=44%, list=17%, signal=53% |
| KEGG_TIGHT_JUNCTION | 132 | -0.52 | -1.98 | 0 | 0.003 | 0.051 | 5441 | tags=36%, list=10%, signal=40% |
| KEGG_APOPTOSIS | 87 | -0.56 | -1.94 | 0 | 0.006 | 0.088 | 7280 | tags=47%, list=13%, signal=54% |
| KEGG_DORSO_VENTRAL_AXIS_FORMATION | 24 | -0.66 | -1.93 | 0.002 | 0.006 | 0.094 | 6601 | tags=54%, list=12%, signal=61% |
| KEGG_LONG_TERM_DEPRESSION | 70 | -0.56 | -1.93 | 0.002 | 0.006 | 0.097 | 7878 | tags=41%, list=14%, signal=48% |
| KEGG_AXON_GUIDANCE | 129 | -0.55 | -1.91 | 0.002 | 0.007 | 0.112 | 9151 | tags=50%, list=17%, signal=59% |
| KEGG_CALCIUM_SIGNALING_PATHWAY | 177 | -0.54 | -1.91 | 0 | 0.007 | 0.112 | 9317 | tags=46%, list=17%, signal=55% |
| KEGG_LEISHMANIA_INFECTION | 70 | -0.67 | -1.91 | 0.014 | 0.007 | 0.114 | 8683 | tags=64%, list=16%, signal=76% |
| KEGG_ERBB_SIGNALING_PATHWAY | 87 | -0.54 | -1.9 | 0 | 0.008 | 0.119 | 8386 | tags=48%, list=15%, signal=57% |
| KEGG_PHOSPHATIDYLINOSITOL_SIGNALING_SYSTEM | 76 | -0.56 | -1.9 | 0.002 | 0.008 | 0.122 | 7394 | tags=46%, list=13%, signal=53% |
| KEGG_INOSITOL_PHOSPHATE_METABOLISM | 54 | -0.59 | -1.9 | 0.002 | 0.008 | 0.122 | 7233 | tags=44%, list=13%, signal=51% |
| KEGG_CHRONIC_MYELOID_LEUKEMIA | 73 | -0.55 | -1.89 | 0.002 | 0.008 | 0.123 | 7674 | tags=48%, list=14%, signal=56% |
| KEGG_NEUROTROPHIN_SIGNALING_PATHWAY | 126 | -0.54 | -1.89 | 0.004 | 0.008 | 0.124 | 8392 | tags=48%, list=15%, signal=57% |
| KEGG_ARRHYTHMOGENIC_RIGHT_VENTRICULAR_CARDIOMYOPATHY_ARVC | 74 | -0.61 | -1.88 | 0 | 0.008 | 0.13 | 7821 | tags=53%, list=14%, signal=61% |
| KEGG_NON_SMALL_CELL_LUNG_CANCER | 54 | -0.55 | -1.88 | 0.008 | 0.008 | 0.133 | 4836 | tags=43%, list=9%, signal=47% |
| KEGG_SPHINGOLIPID_METABOLISM | 39 | -0.59 | -1.88 | 0.004 | 0.008 | 0.136 | 6380 | tags=46%, list=12%, signal=52% |
| KEGG_ENDOMETRIAL_CANCER | 52 | -0.55 | -1.87 | 0.008 | 0.008 | 0.141 | 7268 | tags=50%, list=13%, signal=58% |
| KEGG_GLYCOSPHINGOLIPID_BIOSYNTHESIS_GANGLIO_SERIES | 15 | -0.72 | -1.85 | 0.006 | 0.01 | 0.161 | 6641 | tags=60%, list=12%, signal=68% |
| KEGG_MELANOGENESIS | 101 | -0.52 | -1.85 | 0.002 | 0.01 | 0.163 | 7859 | tags=41%, list=14%, signal=47% |
| KEGG_LONG_TERM_POTENTIATION | 70 | -0.52 | -1.85 | 0 | 0.01 | 0.163 | 6761 | tags=39%, list=12%, signal=44% |
| KEGG_HYPERTROPHIC_CARDIOMYOPATHY_HCM | 83 | -0.57 | -1.83 | 0.004 | 0.011 | 0.179 | 10165 | tags=54%, list=18%, signal=66% |
| KEGG_RIG_I_LIKE_RECEPTOR_SIGNALING_PATHWAY | 70 | -0.51 | -1.83 | 0.004 | 0.011 | 0.179 | 7398 | tags=41%, list=13%, signal=48% |
| KEGG_WNT_SIGNALING_PATHWAY | 151 | -0.48 | -1.83 | 0.002 | 0.011 | 0.179 | 7859 | tags=40%, list=14%, signal=46% |
| KEGG_DILATED_CARDIOMYOPATHY | 90 | -0.57 | -1.83 | 0.004 | 0.011 | 0.18 | 7821 | tags=48%, list=14%, signal=56% |

| | | | | | | | | |
|---|---|---|---|---|---|---|---|---|
| KEGG_VEGF_SIGNALING_PATHWAY | 76 | -0.5 | -1.82 | 0.004 | 0.012 | 0.196 | 6761 | tags=38%, list=12%, signal=43% |
| KEGG_EPITHELIAL_CELL_SIGNALING_IN_HELICOBACTER_PYLORI_INFECTION | 68 | -0.54 | -1.82 | 0.002 | 0.013 | 0.208 | 7280 | tags=43%, list=13%, signal=49% |
| KEGG_VIRAL_MYOCARDITIS | 68 | -0.6 | -1.82 | 0.018 | 0.013 | 0.216 | 11912 | tags=59%, list=22%, signal=75% |
| KEGG_PATHOGENIC_ESCHERICHIA_COLI_INFECTION | 56 | -0.53 | -1.81 | 0.002 | 0.013 | 0.22 | 5977 | tags=39%, list=11%, signal=44% |
| KEGG_SYSTEMIC_LUPUS_ERYTHEMATOSUS | 55 | -0.68 | -1.79 | 0.016 | 0.015 | 0.253 | 10498 | tags=64%, list=19%, signal=78% |
| KEGG_TYPE_II_DIABETES_MELLITUS | 47 | -0.55 | -1.77 | 0.006 | 0.019 | 0.291 | 9255 | tags=45%, list=17%, signal=54% |
| KEGG_HEDGEHOG_SIGNALING_PATHWAY | 56 | -0.55 | -1.76 | 0.006 | 0.019 | 0.293 | 6440 | tags=45%, list=12%, signal=50% |
| KEGG_ETHER_LIPID_METABOLISM | 33 | -0.53 | -1.74 | 0.006 | 0.022 | 0.314 | 4788 | tags=36%, list=9%, signal=40% |
| KEGG_AUTOIMMUNE_THYROID_DISEASE | 50 | -0.6 | -1.73 | 0.046 | 0.024 | 0.335 | 12536 | tags=52%, list=23%, signal=67% |
| KEGG_INTESTINAL_IMMUNE_NETWORK_FOR_IGA_PRODUCTION | 46 | -0.66 | -1.73 | 0.042 | 0.024 | 0.336 | 12934 | tags=74%, list=23%, signal=96% |
| KEGG_GNRH_SIGNALING_PATHWAY | 101 | -0.47 | -1.73 | 0.004 | 0.023 | 0.336 | 6601 | tags=34%, list=12%, signal=38% |
| KEGG_NEUROACTIVE_LIGAND_RECEPTOR_INTERACTION | 271 | -0.51 | -1.73 | 0.002 | 0.024 | 0.344 | 10548 | tags=45%, list=19%, signal=55% |
| KEGG_PROGESTERONE_MEDIATED_OOCYTE_MATURATION | 85 | -0.48 | -1.72 | 0.008 | 0.024 | 0.351 | 4513 | tags=32%, list=8%, signal=35% |
| KEGG_LYSOSOME | 121 | -0.52 | -1.72 | 0.013 | 0.024 | 0.357 | 9474 | tags=46%, list=17%, signal=56% |
| KEGG_O_GLYCAN_BIOSYNTHESIS | 30 | -0.6 | -1.72 | 0.018 | 0.025 | 0.363 | 10391 | tags=50%, list=19%, signal=62% |
| KEGG_PANTOTHENATE_AND_COA_BIOSYNTHESIS | 16 | -0.6 | -1.69 | 0.014 | 0.029 | 0.416 | 5609 | tags=38%, list=10%, signal=42% |
| KEGG_ASTHMA | 28 | -0.69 | -1.68 | 0.042 | 0.032 | 0.446 | 12536 | tags=68%, list=23%, signal=88% |
| KEGG_THYROID_CANCER | 29 | -0.53 | -1.68 | 0.013 | 0.032 | 0.446 | 7657 | tags=45%, list=14%, signal=52% |
| KEGG_INSULIN_SIGNALING_PATHWAY | 137 | -0.43 | -1.67 | 0.006 | 0.034 | 0.461 | 6518 | tags=35%, list=12%, signal=40% |
| KEGG_PRIMARY_IMMUNODEFICIENCY | 35 | -0.63 | -1.65 | 0.043 | 0.038 | 0.493 | 8547 | tags=60%, list=15%, signal=71% |
| KEGG_MTOR_SIGNALING_PATHWAY | 52 | -0.46 | -1.64 | 0.008 | 0.041 | 0.514 | 4660 | tags=31%, list=8%, signal=34% |
| KEGG_STEROID_HORMONE_BIOSYNTHESIS | 55 | -0.51 | -1.62 | 0.026 | 0.044 | 0.533 | 9014 | tags=47%, list=16%, signal=56% |
| KEGG_STARCH_AND_SUCROSE_METABOLISM | 52 | -0.5 | -1.61 | 0.038 | 0.05 | 0.571 | 9014 | tags=42%, list=16%, signal=51% |
| KEGG_ADIPOCYTOKINE_SIGNALING_PATHWAY | 67 | -0.45 | -1.58 | 0.016 | 0.06 | 0.629 | 9833 | tags=37%, list=18%, signal=45% |
| KEGG_DRUG_METABOLISM_CYTOCHROME_P450 | 71 | -0.47 | -1.54 | 0.038 | 0.071 | 0.674 | 13045 | tags=49%, list=24%, signal=64% |
| KEGG_SNARE_INTERACTIONS_IN_VESICULAR_TRANSPORT | 38 | -0.47 | -1.5 | 0.042 | 0.085 | 0.728 | 9140 | tags=45%, list=17%, signal=54% |
| **Gene sets enriched in phenotype C2 (92 samples)** | | | | | | | | |
| KEGG_RNA_POLYMERASE | 29 | 0.65 | 1.9 | 0 | 0.098 | 0.123 | 8151 | tags=62%, list=15%, signal=73% |
| KEGG_RIBOSOME | 88 | 0.84 | 1.83 | 0.008 | 0.107 | 0.213 | 5276 | tags=80%, list=10%, signal=88% |
| KEGG_GLYCINE_SERINE_AND_THREONINE_METABOLISM | 31 | 0.57 | 1.82 | 0.002 | 0.075 | 0.223 | 5266 | tags=52%, list=10%, signal=57% |
| KEGG_BASE_EXCISION_REPAIR | 35 | 0.6 | 1.72 | 0.029 | 0.13 | 0.382 | 3535 | tags=43%, list=6%, signal=46% |
| KEGG_PENTOSE_PHOSPHATE_PATHWAY | 27 | 0.6 | 1.69 | 0.022 | 0.136 | 0.447 | 8324 | tags=52%, list=15%, signal=61% |
| KEGG_GLYOXYLATE_AND_DICARBOXYLATE_METABOLISM | 16 | 0.61 | 1.62 | 0.029 | 0.179 | 0.552 | 4099 | tags=44%, list=7%, signal=47% |
| KEGG_AMINOACYL_TRNA_BIOSYNTHESIS | 22 | 0.62 | 1.59 | 0.048 | 0.186 | 0.606 | 9836 | tags=73%, list=18%, signal=88% |
| KEGG_PYRIMIDINE_METABOLISM | 98 | 0.46 | 1.58 | 0.049 | 0.168 | 0.611 | 8550 | tags=49%, list=15%, signal=58% |
| KEGG_SELENOAMINO_ACID_METABOLISM | 25 | 0.52 | 1.53 | 0.042 | 0.206 | 0.703 | 3865 | tags=40%, list=7%, signal=43% |
