# Supplementary figures and images for: Comprehensive Analysis of Immune Infiltrates of Ferroptosis-Related Long Noncoding RNA and Prediction of Colon Cancer Patient Prognoses
Source: J Immunol Res. 2022 Feb 27;2022:9480628. doi: 10.1155/2022/9480628 (PMC8898846; doi:10.1155/2022/9480628)

A

consensus CDF

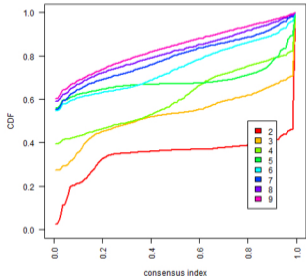

B

Delta area

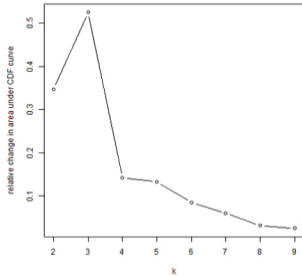

C

tracking plot

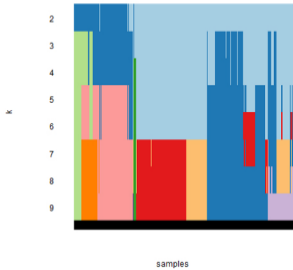

Supplement: Supplementary Materials — Table S1: ferroptosis-related genes downloaded from FerrDb. Table S2: the univariate Cox regression analysis of ferroptosis-related genes. Table S3: characteristics of the two clusters of COAD patients. Table S4: detailed enrichment results of the GSEA in clusters 1 and 2. Figure S1: consensus clustering of the tumorous cohort from TCGA based on differentially expressed ferroptosis-related lncRNAs. Figure S2: distinct pathways enriched in clusters 1 and 2. Figure S3: Fifteen ferroptosis-related lncRNAs were identified via the LASSO regression analysis. [file 9480628.f1.zip › 9480628.f1/Figure S1 (1).pdf]

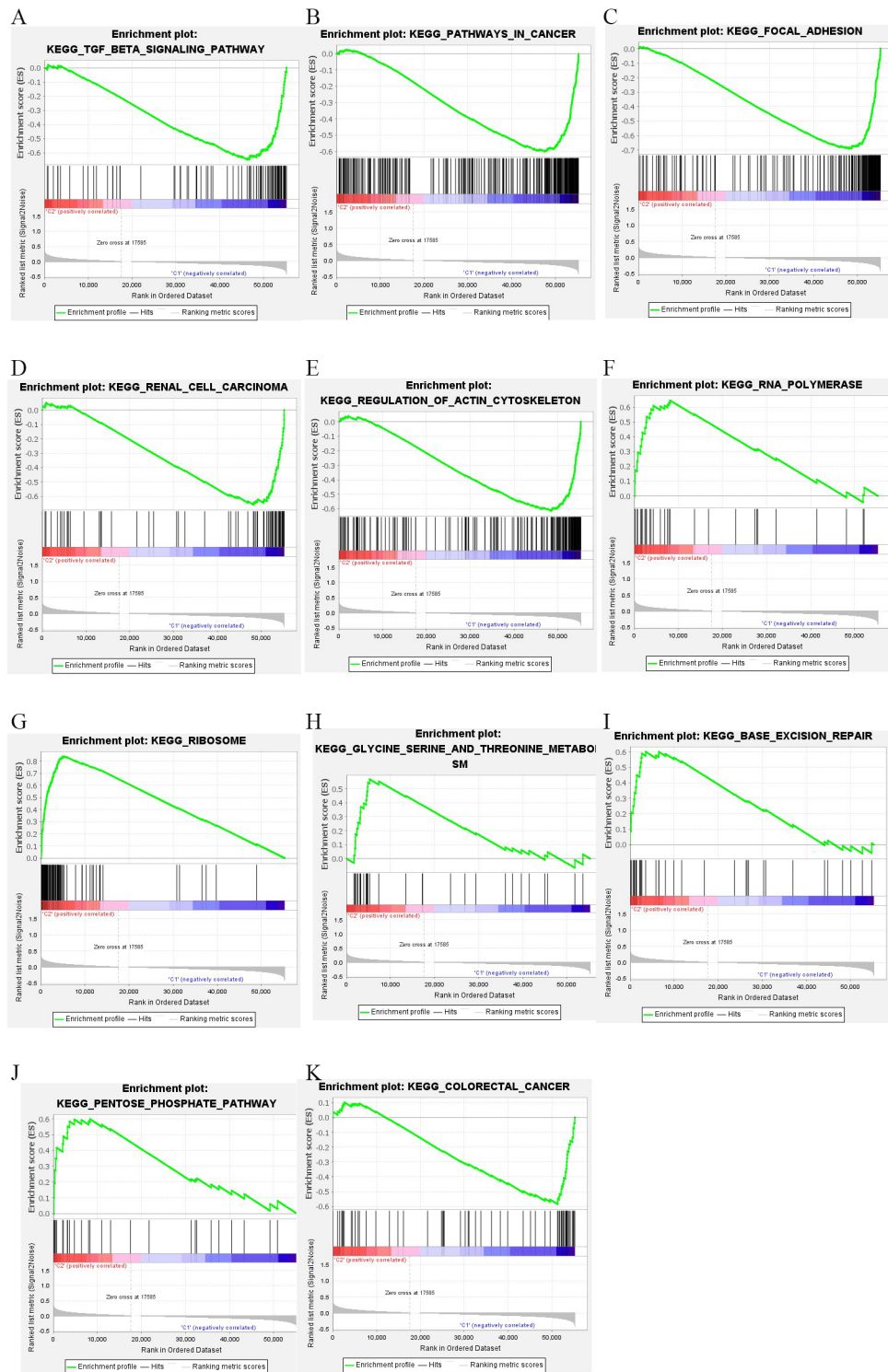

Supplement: Supplementary Materials — Table S1: ferroptosis-related genes downloaded from FerrDb. Table S2: the univariate Cox regression analysis of ferroptosis-related genes. Table S3: characteristics of the two clusters of COAD patients. Table S4: detailed enrichment results of the GSEA in clusters 1 and 2. Figure S1: consensus clustering of the tumorous cohort from TCGA based on differentially expressed ferroptosis-related lncRNAs. Figure S2: distinct pathways enriched in clusters 1 and 2. Figure S3: Fifteen ferroptosis-related lncRNAs were identified via the LASSO regression analysis. [file 9480628.f1.zip › 9480628.f1/Figure S2 (1).pdf]

A

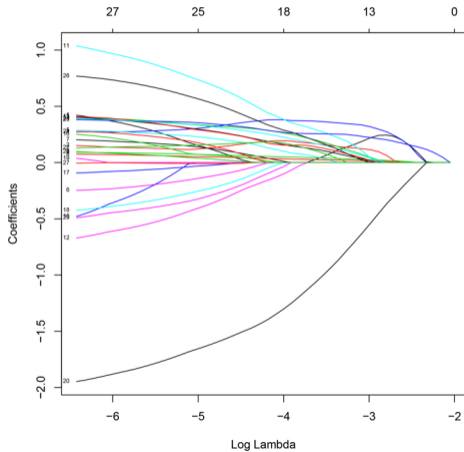

B

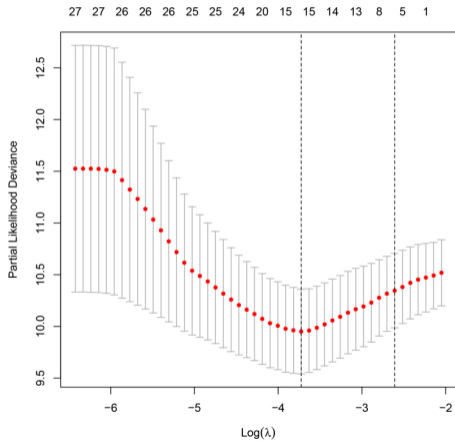

Supplement: Supplementary Materials — Table S1: ferroptosis-related genes downloaded from FerrDb. Table S2: the univariate Cox regression analysis of ferroptosis-related genes. Table S3: characteristics of the two clusters of COAD patients. Table S4: detailed enrichment results of the GSEA in clusters 1 and 2. Figure S1: consensus clustering of the tumorous cohort from TCGA based on differentially expressed ferroptosis-related lncRNAs. Figure S2: distinct pathways enriched in clusters 1 and 2. Figure S3: Fifteen ferroptosis-related lncRNAs were identified via the LASSO regression analysis. [file 9480628.f1.zip › 9480628.f1/Figure S3 (1).pdf]
